# Combined pigmentary and structural effects tune wing scale coloration to color vision in the swallowtail butterfly *Papilio xuthus*

**DOI:** 10.1186/s40851-015-0015-2

**Published:** 2015-04-24

**Authors:** Doekele G Stavenga, Atsuko Matsushita, Kentaro Arikawa

**Affiliations:** Computational Physics, Zernike Institute for Advanced Materials, University of Groningen, Groningen, NL9747AG The Netherlands; Laboratory of Neuroethology, SOKENDAI (The Graduate University for Advanced Studies), Hayama, 240-0115 Japan

**Keywords:** Papiliochrome, Melanin, Structural coloration, Interference reflector, Optical multilayer

## Abstract

Butterflies have well-developed color vision, presumably optimally tuned to the detection of conspecifics by their wing coloration. Here we investigated the pigmentary and structural basis of the wing colors in the Japanese yellow swallowtail butterfly, *Papilio xuthus*, applying spectrophotometry, scatterometry, light and electron microscopy, and optical modeling. The about flat lower lamina of the wing scales plays a crucial role in wing coloration. In the cream, orange and black scales, the lower lamina is a thin film with thickness characteristically depending on the scale type. The thin film acts as an interference reflector, causing a structural color that is spectrally filtered by the scale’s pigment. In the cream and orange scales, papiliochrome pigment is concentrated in the ridges and crossribs of the elaborate upper lamina. In the black scales the upper lamina contains melanin. The blue scales are unpigmented and their structure differs strongly from those of the pigmented scales. The distinct blue color is created by the combination of an optical multilayer in the lower lamina and a fine-structured upper lamina. The structural and pigmentary scale properties are spectrally closely related, suggesting that they are under genetic control of the same key enzymes. The wing reflectance spectra resulting from the tapestry of scales are well discriminable by the *Papilio* color vision system.

## Introduction

Butterflies are highly visual animals, with well-developed color vision systems. The butterfly species whose visual capacities have so far been investigated into the most detail is the Japanese yellow swallowtail butterfly, *Papilio xuthus*. By combining anatomy and intracellular electrophysiology with spectrophotometry and related optical methods, the set of retinal photoreceptors has been shown to consist of at least six different spectral classes, sensitive in the ultraviolet (UV), violet, blue, green, red and broad-band wavelength regions, respectively [1,2]. Additional behavioral investigations have revealed the butterfly’s extreme wavelength discrimination capacities. *P. xuthus* color vision appears to be tetrachromatic, based on a set of UV, blue, green and red receptors [3].

Butterflies use their color vision to search for flower nectar and presumably for detecting and recognizing conspecifics [2]. We therefore considered that the wing coloration of *P. xuthus* might be tuned to its color vision system. This initiated an optical analysis of the wing scales that cover the wing substrate, so together forming a tapestry creating a colorful and flashing signal displayed during flight [4]. We recall here that the basic bauplan of a butterfly wing scale consists of two laminae. The lower lamina is a more or less flat plate, connected by trabeculae to the elaborate array of parallel ridges of the upper lamina, which are connected by crossribs. Generally the latter form open windows to the scale lumen, but when a membrane connects the crossribs, the windows are closed [5,6].

The vast majority of butterfly wing scales contains pigments, with melanin being most universal, causing brown and often very black scales [4]. The characteristic color pigments of papilionid butterflies belong to the class of papiliochromes [7]. For instance, the pigment of the cream scales of *P. xuthus*, which absorbs exclusively in the ultraviolet to violet wavelength region, has been characterized as papiliochrome II, and the pigment of the orange scales, which absorbs in a broader wavelength range, is presumably a related papiliochrome pigment [8]. Interestingly, females of the Eastern tiger swallowtail (*Papilio glaucus*) of North America can be either yellow (wild type) or black (melanic) [9,10]. The melanic form is a Batesian mimic of the distasteful Pipevine swallowtail (*Battus philenor*), which is also black in overall color. In melanic females melanin replaces the background yellow. The key enzyme involved is N-b-alanyl-dopamine-synthase (BAS), which shunts dopamine from the melanin pathway into the production of the cream-yellow coloring papiliochrome. This enzyme is suppressed in melanic females, leading to abnormal melanization of a formerly yellow area, and wing scale maturation is also delayed in the same area. The changes in expression of a single gene product thus result in multiple wing color phenotypes [9,10].

It is commonly assumed that the color of pigmented butterfly scales is caused by the irregular structures of the upper lamina acting as random scatterers and that the contained pigment determines the scale’s color by selective spectral filtering of the scattered light. However, notably in scales with open windows, a considerable fraction of incident light will pass the ridges and crossribs and thus reach the lower lamina. It will be there reflected dependent on the lower lamina’s structural properties. The reflected light flux will partly leave the scale unhampered through the windows, but it will also partly traverse the ridges and crossribs of the upper lamina, where it will be spectrally filtered by the pigments present.

Scales that do not harbor pigment can still be very colorful when they have regularly arranged, nano-sized structures. Optical interference, which enhances light reflection in a restricted wavelength range and suppresses the reflection in the complementary wavelength range, then creates distinct colors [11,12]. The iconic examples with structural coloration are the *Morpho* butterflies, which have scales with ridges exquisitely structured into optical multilayers [5,6,13,14]. Among the papilionids, the Cattlehearts (*Parides* species) have scales furnished with multilayers or gyroid photonic crystals [8,13,15].

In the last decades many studies have been devoted to unraveling the complex optics of the photonic structures of butterflies, but it was only very recently recognized that in scales with a single-layered lower lamina optical interference may also play a crucial role in scale coloration. Research on the wing scales of *B. philenor* and a number of nymphaline butterfly species indicated that the lower lamina acts as an optical thin film, reflecting predominantly light in a limited wavelength range [16,17]. In other words, the reflectance spectrum of a butterfly wing scale can be principally determined by the pigmentation, alternatively by wave-optical effects, or by a combination of the pigmentary and structural scale properties [16-18].

In the common butterfly wing scale the lower lamina is a thin film. Because a thin film’s thickness primarily determines its reflectance spectrum, the direct inference of the previous studies was that the differently colored wing scales of butterflies may have lower laminae with different thicknesses. If so, the thickness of the lower lamina must then be under genetic control and hence tunable and subject to evolutionary pressure. To substantiate the role of the lower lamina in butterfly wing scale coloration and to investigate the possible tuning of scale color and color vision, we chose *P. xuthus* for a detailed investigation of the structural and optical characteristics of the variously colored wing scales. To be able to directly assess the lower lamina’s thickness we performed anatomy, and this indeed yielded substantially different thickness values for the various scales. To understand the scales’ different coloring methods into quantitative detail, we applied spectrophotometry and scatterometry on single wing scales and performed optical modeling. The obtained results reinforced our view that pigmentation and scale structure are intimately connected and furthermore caused the hypothesis that the wing colors are tuned to the swallowtail’s color vision system.

## Materials and methods

### Animals

Specimens of the Japanese yellow swallowtail butterfly, *Papilio xuthus*, were from a laboratory culture derived from eggs laid by females caught in Kanagawa, Japan. Photographs of a mounted specimen were taken with a Nikon D70 digital camera equipped with an F Micro-Nikkor (60 mm, f2.8; Nikon, Tokyo, Japan) macro objective.

### Scale photography

Photographs of single scales, obtained by gently pressing the wings onto a microscope slide, in immersion fluid were made with a Zeiss Universal Microscope, using epi-illumination through a Zeiss Epiplan 16x/0.35 objective (Zeiss, Oberkochen, Germany). Photographs were also taken of both sides, abwing and adwing, of single scales glued to a glass micropipette. The adwing (or under) side faces the wing substrate *in situ*, and the abwing (or upper) side is the exposed side that is seen when observing the scales on the wing.

### Anatomy

For light and electron microscopy of the scales’ anatomy, wing parts were prefixed in 2% paraformaldehyde and 2.5% glutaraldehyde in 0.1 mol l^−1^ sodium cacodylate buffer (CB, pH 7.3) or sodium phosphate buffer (PB, pH 7.3) for ~45 min at 20–25°C. Embedding in Spurr followed dehydration with a graded series of ethanol and infiltration with propylene oxide. For light microscopy, the tissues were cut into ~1 μm sections and observed with a BX51 (Olympus) microscope, equipped with a DP71 (Olympus) camera system. For transmission electron microscopy, the tissues were cut into 50 nm sections, double-stained with uranyl acetate and lead nitrate, and observed with an H7650 transmission electron microscope (Hitachi, Tokyo, Japan), equipped with a CCD camera (Morada, Seika Digital Image Corp., Tokyo, Japan). For scanning electron microscopy, the wing pieces were sputtered with platinum and observed with a JSM-6490LV (JEOL, Tokyo, Japan).

### Spectroscopy

Reflectance spectra of single wing scales *in situ* or from isolated scales taken from different wing areas and attached to a glass micropipette were measured with a microspectrophotometer (MSP), being a Leitz Ortholux microscope (Leitz, Wetzlar, Germany) connected to an AvaSpec 2048–2 CCD detector array spectrometer (Avantes, Eerbeek, Netherlands), with light supplied by a xenon arc light source. The microscope objective was an Olympus LUCPlanFL N 20x/0.45 (Olympus, Tokyo, Japan). A white diffuse reflectance tile (Avantes WS-2) was used as a reference. Absorbance spectra of single scales, immersed in immersion oil or water were also measured with the MSP. Reflectance measurements of various wing areas of intact butterfly wings were measured with a bifurcated probe (Avantes FCR-7UV200; Avantes, Eerbeek, Netherlands); the light source was a deuterium-halogen lamp (AvaLight-D(H)-S), and the white diffuse reflectance tile served as reference. It is important to note that the diffuse reference yields for specular objects reflectance spectra where the shape is representative but the amplitude is overestimated.

**Figure 1:**
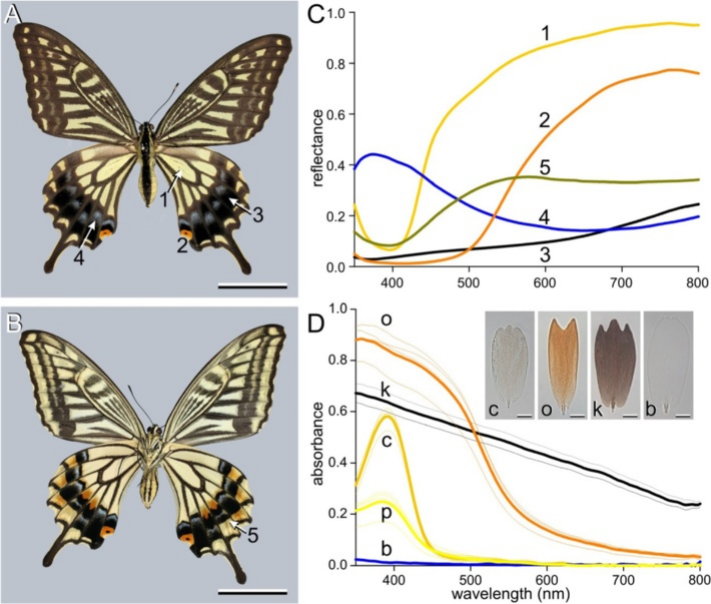
Wing coloration of *Papilio xuthus*.** A**, **B** Dorsal and ventral view of a spring form female. The numbers refer to the wing areas from where scales were taken (scale bar: 2 cm). **C** Reflectance spectra measured with a microspectrophotometer from single scales *in situ* of the numbered areas of **A** and **B**. **D** Absorbance spectra of scales embedded in immersion oil; c: a cream scale (from area 1); o: orange scale (area 2); k: black scale (area 3), b: blue scale (area 4); scales of area 5 are not included (scale bar: 20 μm). The thin curves are from small measurement areas (15 × 15 μm^2^) near the tip and middle parts of a few different scales. The bold curves represent the averaged spectra (of 4, except for the black: 2). The deep-yellow curves are from cream-colored (c) scales on the dorsal hindwing, from area 1, and the yellow spectra are from pale-cream-colored (p) scales located on the ventral hindwing, opposite to the dorsal area 1.

### Imaging scatterometry

The spatial reflection characteristics of the wing scales were studied with an imaging scatterometer. An isolated, single scale attached to a glass micropipette was positioned at the first focal point of the ellipsoidal mirror of the imaging scatterometer [19]. The scatterograms were obtained by focusing a white light beam with narrow aperture (<5°) onto at a small circular area (diameter ~13 μm) of the scale, and the spatial distribution of the far-field scattered light was then monitored. A flake of magnesium oxide served as a white diffuse reference object (for further details, see [19]).

### Modeling of reflectance spectra

The reflectance spectrum of the lower lamina of the pigmented scales was calculated for normally incident light using the classical Airy formulae for thin films [18,20], assuming that the scale’s lower lamina acts as an optical thin film with thickness depending on the scale type. The reflectance spectrum of the lower lamina of blue scales was modeled by treating it as an optical multilayer, using a matrix-transfer formalism [18].

## Results

### Wing colors and pigmentation

The wing colors of *P. xuthus* can be distinguished into the following main categories: yellow, orange, black, and blue (numbered 1–4 in Figure 1A). These colors are observable on both the dorsal (Figure 1A) and the ventral wings (Figure 1B). A rare, fifth scale category with a yellow-ochre color exists at the ventral hindwings (#5, Figure 1B). Because the colors originate from the lattice of wing scales, as a first characterization we measured reflectance spectra from individual scales in the differently colored areas (Figure 1C).

The spectra suggested the presence of various pigments, and to investigate this more closely we picked up scales from the wing and measured the absorbance spectra of single scales with a microspectrophotometer (Figure 1D). To suppress possible structural colorations, we immersed the scales in immersion oil. The single scales obtained from the dorsal yellow wing areas (Figure 1A, #1) had an unsaturated yellow, cream color. They prominently absorbed only in the ultraviolet to violet wavelength range, with peak absorbance of ~0.6 (c in Figure 1D). The responsible pigment has been characterized as papiliochrome II [7]. Scales of the ventral yellow wing areas (Figure 1B) had a much paler cream color. Their absorbance spectrum had the same shape as that of the dorsal cream scales, but the peak absorbance was no more than ~0.2 (p in Figure 1D), meaning that the density of the papiliochrome II pigment in the ventral scales was distinctly lower than that in the dorsal scales. The scales obtained from the orange wing areas absorbed well into the visible wavelength range and showed considerable peak absorbances, approaching 1 (Figure 1A, #2; o in Figure 1D). The absorbance of single black scales was substantial throughout a very broad wavelength range, clearly due to high concentrations of melanin pigment (Figure 1A, #3; k in Figure 1D). Very differently, the blue scales had a negligible absorbance (Figure 1A, #4; b in Figure 1D), meaning that the blue color must have a structural origin.

### Structure of the wing scales of *P. xuthus*

**Figure 2:**
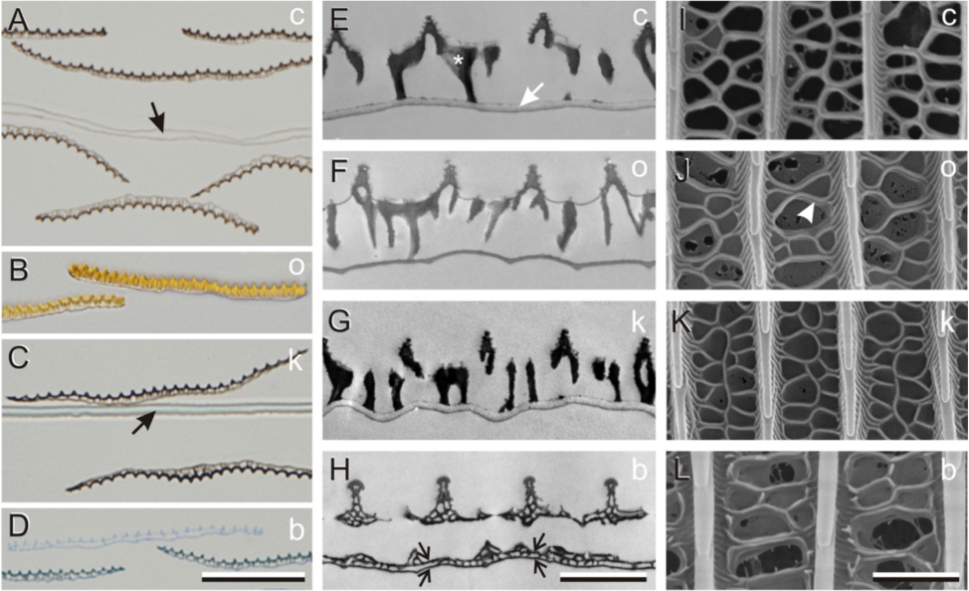
Anatomy of cream (c), orange (o), black (k), and blue (b) wing scales. **A**-**D** Light microscopy. **E**-**H** Transmission electron microscopy. **I**-**L** Scanning electron microscopy. In **A** and **C** the wing membrane substrate (arrows) has on both sides cream and black scales, respectively. The section of **D** was stained with Azur II to visualize the unpigmented blue scale, which is situated above two black scales. The upper lamina has in **E** (asterisk) and **G** a high electron density, but in the lower lamina (**E**, white arrow) the electron density is low. The lower lamina of the blue scales consists of two membranes (**H**, arrows) with local connections. Membranous structures can be seen between crossribs (e.g., arrowhead in **J**). Scale bars: **A**-**D** 20 μm. **E**-**L** 2 μm.

**Figure 3:**
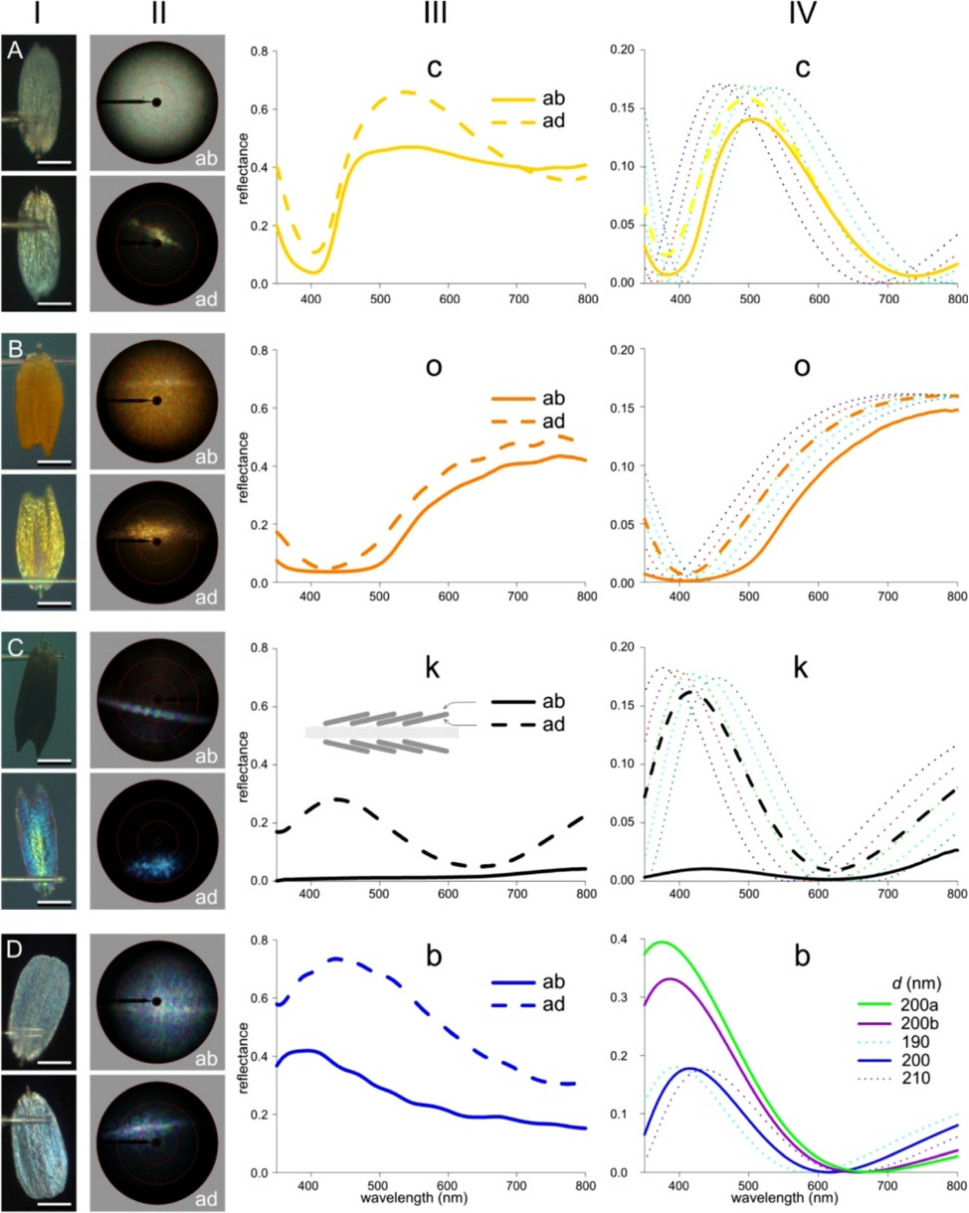
Photographs, scatterograms, and reflectance spectra of single scales, glued to a thin glass micropipette, with modeled spectra. **A**-**D** A cream (c), orange (o), black (k), and blue (b) scale, respectively. In each panel, photographs (column I) and scatterograms (column II) of the upper and lower side (abwing: ab, and adwing: ad; see inset **C**, column III) of the scales are shown above and below, respectively. The red circles in the scatterograms indicate scattering angles of 5°, 30°, 60°, and 90°. The reflectance spectra were measured with respect to a diffusely reflecting white reference, and thus yielded too high values for directionally reflecting media. **A**-**C**, column IV, present reflectance spectra modeled with optical thin film and multilayer theory for five thin films consisting of butterfly chitin in air with different thicknesses (dotted curves) and their average (dashed bold curve), for the cream (c), orange (o), and black (k) scales, respectively. The averaged spectra, when multiplied with a spectrum representative for the transmittance spectrum of the scale’s pigment (derived from Figure 1D) yielded the continuous bold curves. The thickness (in nm) of the five scales was: 210 + 10*i* (c), 115 + 5*i* (o), 170 + 10*i* (k), with *i* =1-5. **D** column IV, presents reflectance spectra of three chitinous thin films in air with thickness 190, 200, and 210 nm, and reflectance spectra of a stack of three parallel layers in air, with thicknesses 60, 80 and 60 nm, where the upper and lower layer had the refractive index of chitin. The refractive index of the middle layer was taken to be a weighted average of the refractive indices of chitin and air with ratio 1:2 (200a) and ratio 1:1 (200b).

Whereas the color of the blue scales must be fully structural, the color of the pigmented scales might not be fully determined by the pigment and some structural coloration could be present also. To clarify the possible contribution of the scales’ structures to their color, we investigated the anatomy of the scales. As can be directly observed with a binocular microscope, the scales exist on both sides of the wing and partly overlap each other, and this causes the partly stacked scales in the light microscopical cross-sections of the various wing parts carrying the four main scale types (Figure 2A-D). The light micrographs showed that the scales of *P. xuthus* consist of a more or less flat lower lamina, which faces the wing substrate, and a highly structured upper lamina. The lower and upper lamina are therefore also called the adwing and abwing lamina, respectively (latin ad: to, on; ab: away from; see inset Figure 3C, column III). The cream, orange and black scales appeared to have their dense pigmentation concentrated in the upper lamina, which made them easily recognizable in unstained sections (Figure 2A-C). The pigment concentration in the blue scales was too low for recognition of the scales, however, and therefore the section of Figure 2D, showing a blue scale with two underlying black scales, was stained with Azur II.

Transmission electron micrographs of the pigmented scale types yielded very similar structures (Figure 2E-G). Quite strikingly, the thickness of the lower lamina varied among these scales, with notably the orange scales having a very thin lower lamina (Figure 2F). The electron micrographs of all pigmented scales showed much less contrastful lower laminae than the elaborately structured upper laminae, in agreement with the predominant or even exclusive presence of pigment in the upper laminae (Figure 2E-G). Surprisingly, the blue scales showed a very aberrant anatomy. Both the lower and upper lamina appeared to have a somewhat similar holey structure, with the lower lamina essentially consisting of two membranes, which were only locally connected (Figure 2H). The lower lamina of the blue scale thus is not a thin film but rather a multilayer.

Scanning electron microscopy of the upper lamina of the various scales showed in all cases a structure characteristic for papilionid butterflies (Figure 2I-L). The parallel ridges, with interdistance 1–2 μm, were connected with irregularly arranged crossribs, with often closed windows, that is, connected by a membrane. Taken together, the anatomical studies showed that pigment, if present, is at least for the major part expressed in the upper lamina, that the lower lamina of the pigmented scales is basically a thin film and thus will act as an interference reflector, and that structural effects determine the color of the blue scales.

### Spatial and spectral characteristics of the scale reflections

To further investigate the contribution of the scale structures to the scale coloration, we applied imaging scatterometry and microspectrophotometry on both the abwing (upper) side and adwing (under) side of single scales. The photographs and scatterograms of the investigated scales (columns I and II of Figure 3) indicated that the optical properties of the two opposite sides of the scale rather differ. The abwing side was always matte. Accordingly, scatterometry using narrow-beam illumination produced wide-angled, diffuse scatterograms; in most cases also a narrow, colored line appeared, revealing diffraction on the array of parallel ridges (Figure 3B-D, column II, ab). The adwing side of the scales was generally rather glossy, indicating that the lower lamina acted mirrorlike, and the corresponding scatterograms accordingly showed a spatially restricted pattern. If the lower laminae had been perfect thin films, the scatterograms would have shown single dots, but the patterns were in fact slightly blurred. Indeed, the photographs of the adwing side (Figure 3A-D, column I, ad) indicated that the lower lamina of the scales was not perfectly flat but slightly ripply (see also Figure 2A-H).

The different scale types had each very specific reflectance spectra (Figure 3A-D, column III). The scatterograms of the adwing side of the cream, orange and black scales indicate that the lower lamina’s thin film reflections must determine the adwing reflectance spectrum. On the other hand, the reflectance spectrum of their abwing side may be expected to be greatly determined by the absorbance spectra of the various pigments. Strikingly, however, the reflectance spectra of both sides of the cream and orange scales were similar, indicating that thin film reflectance and pigment absorption are spectrally tuned. In the black scales the reflectance of the abwing side was very minor throughout the whole ultraviolet and visible wavelength range, clearly due to the strongly absorbing melanin (Figure 1D). Nevertheless, the adwing reflectance spectrum of the black scales featured a distinct, blue-green peaking spectrum, very characteristic of an optical thin film. In fact, all adwing reflectance spectra of Figure 3 suggested the presence of an interference reflector. To substantiate this we performed optical modeling, using the measured pigment spectra and the anatomy of the four main scale types.

### Modeling scale reflectance spectra

The transmission electron micrographs of the cream, orange and black scales showed that their lower lamina was a single layer with thickness between 100 and 250 nm. Using classical thin film theory we calculated reflectance spectra for the lower lamina of the different scales (Figure 3A-C, column IV). We assumed the absence of pigment in the lower lamina, as suggested by both the light and transmission-electron micrographs, and we took for the refractive index of the lower lamina’s material, chitin, the value determined for the glass scales of *Graphium sarpedon* [21]. The TEM micrographs (Figure 2E-G) showed that the scale thicknesses were not constant, and therefore we calculated for each scale type reflectance spectra for five values of the thickness, *d*; for the cream scale *d* = 220, 230, …260 nm (Figure 3A, column IV), for the orange scale *d* = 120, 125 …140 nm (Figure 3B, column IV), and for the black scale *d* = 180, 190,… 220 nm (Figure 3C, column IV). For each scale type we averaged the five reflectance spectra (Figure 3A-C, column IV, dotted curves), which yielded for the cream scale a distinctly blue-green peaking spectrum (at ~500 nm), for the orange scale an increasing reflectance in the visible wavelength range, and for the black scale a blue-peaking reflectance, with maximum near 410 nm (Figure 3A-C, column IV, dashed curves). The averaged spectra resembled the measured reflectance spectra of the adwing sides of the scales, but a close correspondence was not reached, except perhaps for the black scale. An obvious reason for the discrepancy is that in the calculations only the thin film of the lower lamina was taken into account, while the other scale components may also contribute to the adwing reflectance spectra. Especially in the longer wavelength range, where pigment absorption is minor, background scattering from the upper lamina will raise the reflectance.

For the adwing reflectance spectrum of the blue scales, the situation is even more complex. The transmission electron micrograph of Figure 2H showed that the lower lamina is not a single thin film, but rather a multilayer, consisting of two thin membranes separated by an air gap, with pieces of membrane that connect the two outer layers. The total thickness of the lower lamina was about 200 nm, with thickness of the two membranous outer layers ~60 nm and that of the middle layer, consisting mostly of air, ~80 nm. To calculate the reflectance spectrum of this structure, we assumed that the two membranes consisted of chitin and that the refractive index of the middle layer is the weighted average of chitin and air. We considered two cases, with the membrane to air ratio of the middle layer being 1:2 and 1:1, respectively. Using a matrix-transfer procedure for optical multilayers we calculated that the reflectance spectrum of the three-layered lower lamina has a distinct peak near 400 nm, with amplitude increasing with decreasing membrane content (Figure 3D, column IV; for comparison we have included the reflectance spectra of single thin films consisting of chitin with thicknesses 190, 200, and 210 nm). Clearly, the splitting of a solid thin film into three layers (chitin-air-chitin) causes, in addition to a slight shift in peak wavelength, a most substantial increase in reflectance (note the difference in ordinate in Figure 3D column IV with that of Figure 3A-C column IV).

For the interpretation of the reflectance spectra measured from the abwing side of the various scale types, the properties of the upper lamina have to be included. However, the structure of the upper lamina is too complex for a simple computational treatment. The main effect of the highly convoluted upper lamina will be a diffuse, more or less wavelength-independent reflection, which is spectrally filtered by the expressed pigment. In the cream, orange and black scales, incident light that has traversed the upper lamina and is reflected by the lower lamina will be spectrally filtered by the pigment in the upper lamina. We have assessed the spectral filtering for each scale type by multiplying the averaged thin film reflectance spectra of Figure 3A-C (column IV, dashed curves) with the transmittance spectrum following from the absorbance spectrum of the scale’s pigment (Figure 1D). The resulting spectra showed a slightly suppressed reflectance for the cream and orange scales and a substantially reduced reflectance for the black scales (Figure 3A-C, column IV, continuous curves). We have to emphasize that the latter spectra are only one component of the reflectance spectrum measured from the abwing side. Another component is the direct (spectrally filtered) backscattering by the ridges and crossribs. Furthermore, when the windows are closed, the membrane between the crossribs will locally also act as a thin film reflector and thus add to the reflectance in a wavelength-dependent manner.

### Wing coloration and color vision

**Figure 4:**
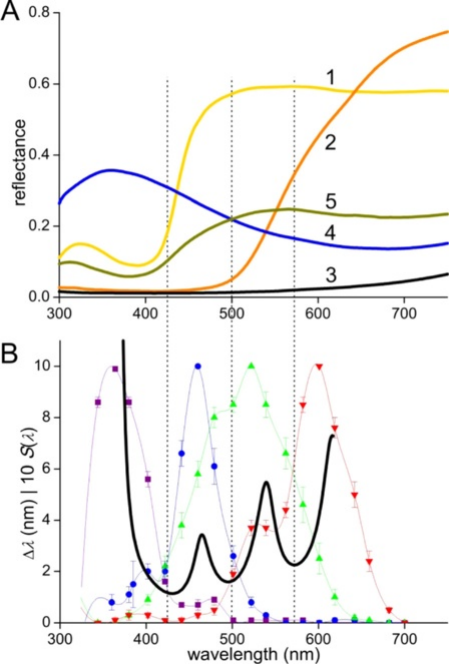
Wing coloration and color discrimination of *Papilio xuthus*.** A** Reflectance spectra measured with a bifurcated probe from wing areas 1–5. **B** Wavelength discrimination as a function of wavelength, ∆λ(λ), following from behavioral experiments (after Figures 1b and 4d of [3]) and the spectral sensitivity, *S*(λ), measured by intracellular recordings, of the four photoreceptor types that were concluded to participate in spectral discrimination; the normalized *S*(λ)-values were multiplied with a factor 10 to facilitate visualization. The dotted vertical lines, with interdistance 80 nm, are at the wavelengths where ∆λ was about minimal.

We have found that the wings of *P. xuthus* has scales with a restricted set of colors. Extensive research demonstrated that the eyes are furnished with a restricted set of spectral photoreceptors. We therefore conjectured that the wing colors are intimately related to the spectral sensitivities of the photoreceptors. To test this hypothesis, we have measured reflectance spectra of the various colored wing areas, using a bifurcated probe (Figure 4A), and compared the spectra with wavelength discrimination (∆λ) data obtained from behavioral studies [3]. The latter data showed three wavelengths where wavelength discrimination was minimal: ~420, 500 and 580 nm (Figure 4B). The minima separate the visible spectrum of *P. xuthus* into four wavelength regions where the four photoreceptors, assumed to form the basis of the spectral discrimination system, are maximally sensitive, i.e. the ultraviolet (UV), blue (B), green (G), and red (R) sensitive photoreceptors. Comparing Figure 4A and 4B suggests that the wing colors are well discriminable by the color vision system.

## Discussion

### Wing scale colors are due to spectrally tuned pigment filters and interference reflectors

The wings of the Japanese yellow swallowtail *P. xuthus* butterfly are colored by scales that contain various pigments and consist of nanostructured elements (Figures 1 and 2). Due to their wavelength-selective absorption, pigments generally play an important role in coloration. It hence has been commonly assumed that the color of the cream and orange scales is principally caused by their specific papiliochromes, especially because the abwing and adwing side of the scales are similarly colored (Figure 3A, B, column I), and indeed the reflectance spectra measured from both scale sides differ only slightly (Figure 3A, B, column III). However, the scatterograms demonstrate that the lower lamina acts as a thin film interference reflector (Figure 3A, B, column II). The remarkable finding that the pigmentation of the upper lamina and the thin film optics of the lower lamina yield very similar reflectance spectra forces the conclusion that the two different optical mechanisms are spectrally tuned.

Optimal spectral tuning can even be argued for the black scales, which contain the broad-band absorbing melanin. Adwing illumination yielded a blue-peaking reflectance spectrum (Figure 3C, column III), which makes sense, because with abwing illumination part of the incident light will reach the lower lamina. The resulting blue-peaking reflectance will be effectively suppressed by the melanin in the upper lamina, because melanin absorbs more strongly at shorter than at longer wavelengths (Figure 1D). Hence a blacker scale is realized when the lower lamina reflects maximally in the blue rather than at longer wavelengths (Figure 3C, column III).

The reflectance spectrum of the blue scales shows a shallow valley in the long-wavelength range, similarly as the rare yellow-ochre scales (Figure 1C #4, 5). Presumably the ultrastructure of the blue and yellow-ochre scales is similar, but the low reflectance around 400 nm of the latter scales indicates that they contain papiliochrome II.

### Genetic control of scale pigment and structure

The expression of pigments in the wing scales has been studied in a few related papilionids. In the Common Mormon swallowtail butterfly, *Papilio polytes*, whose females mimic the unpalatable Common Rose swallowtail butterfly, *Pachliopta aristochiae*, the pale-yellow wing regions in non-mimetic females consist of kynurenine and N-b-alanyldopamine (NBAD). The kynurenine/NBAD biosynthetic genes are absent in mimitic females [22]. Furthermore, Nishikawa et al. [22] found that kynurenine/NBAD synthesis and *Toll* signaling genes were upregulated in the red spots specific to mimetic female wings. In the Eastern tiger swallowtail, *Papilio glaucus*, the wild type females are yellow, but the black, melanic form is a Batesian mimic of the distasteful Pipevine swallowtail (*B. philenor*), which has an overall black color [9,10]. In melanic females, N-b-alanyl-dopamine-synthase (BAS) is suppressed, so that melanin replaces papiliochrome II.

As in the present study on *P. xuthus* scales, in our previous study on *B. philenor* wing scales [16] we found a clear correlation of the ad- and abwing reflectance spectra of the various colored wing scales, reinforcing the hypothesis that both scale structure (and thus lower lamina thickness) and pigment expression are under genetic control of the same key enzymes. Similar conclusions were drawn for the wing scales of a few nymphaline butterfly species, as there the absorbance spectra of the pigment in the upper lamina and the reflectance spectra of the lower lamina appeared to be tuned also [17]. The latter spectra could be well described by thin film modeling, and therefore the lower lamina of the scales of the nymphalines were assumed to be a single layer. However, detailed transmission electron microscopy was not performed in the earlier studies. The present anatomy confirmed that the lower laminae of the pigmented scales of *P. xuthus* were single layers, but surprisingly the lower lamina of the blue scales deviated from a single thin film and appeared to be a multilayer. The anatomy of the scales of other butterfly species therefore deserves to be examined more closely.

An architecture very similar to that of the blue scales of *P. xuthus*, but even more elaborate, exists in the blue scales of lycaenids, the blues [23-26]. In these scales, the lumen is filled with several evenly spaced membranes, connected by minor struts, that together create a brightly reflecting multilayer. Another method of creating bright blue reflectors is the extensive folding into multilayers of the ridges found in *Morpho* butterflies [5,6,13,24].

### The wing scale colors are tuned to the butterfly’s color vision

The coloration of the wing scales of *P. xuthus* appears to be optimized in different ways. The unpigmented blue scales have an increased blue-peaking reflectance due to an increased number of layers in the lower lamina. Also the upper lamina is fine-structured (Figure 2H). The various papiliochromes are expressed in scales with a lower lamina that reflects maximally in the wavelength range where pigment absorption is minimal, suggesting that the structural properties of the lower lamina and the pigmentation are under the same genetic control. Very recently, Monteiro and colleagues demonstrated in the satyrine butterfly *Bicyclus anynana* that the thickness of the lower lamina of certain scales can be specifically modified in a few generations by selectively breeding butterflies with the scale color as selection criterion [27]. Presumably therefore, the restricted set of wing colors of *P. xuthus* is the result of evolutionary selection processes.

The obvious question, what drives the tuning of the scale colors, suggests an obvious answer, namely selection by intraspecific visual recognition. Figure 4 indicates that the yellow wing areas excite the UV receptor to a minor extent and the B, G and R receptors strongly, while the orange wing parts negligibly stimulate the UV and B receptors and much more the G and R receptors; the blue wing spots do the opposite. *P. xuthus* lacks red scales, present in many other papilionid butterflies, which presumably contain another papiliochrome pigment [8,22]. If papilionid butterflies have similar color vision capacities, the red wing areas will stimulate virtually exclusively the red receptors.

Butterfly wings are covered by a tapestry of overlapping scales and therefore the color of the various wing areas is the result of a summation of reflections on the stacks of wing scales on both sides of the wing together with the wing substrate proper (Figure 2A-C). Consequently, the reflectance spectra are much higher than those obtained from the individual scales. For instance, the stacked cream scales create a yellowish color, which is more saturated than that of single scales (Figures 3 and 4). This does not hold for the blue scales, however, because they are stacked upon black scales (Figure 2D) as otherwise the blue structural color would become desaturated [16,17].

When mating, male butterflies find females due to the yellow and black areas, which create a characteristic banding pattern with high color as well as brightness contrast [28]. Interestingly, the most colorful patches are on the lower region of the hindwings on both wing sides (Figure 1A,B). This region is not covered when sitting, neither with open nor with closed wings. Even when the butterflies forage on flowers, the hindwings do not move much compared to the forewings. As the color patterns of both forewings and hindwings are generally well displayed they will thus serve for conspecifics as a conspicuous recognition signal, allowing optimal detection, thanks to well-tuned scale colors.

## References

[1] Arikawa K (2003). Spectral organization of the eye of a butterfly, *Papilio*. J Comp Physiol A.

[2] Kinoshita M, Arikawa K (2014). Color and polarization vision in foraging *Papilio*. J Comp Physiol A.

[3] Koshitaka H, Kinoshita M, Vorobyev M, Arikawa K (2008). Tetrachromacy in a butterfly that has eight varieties of spectral receptors. Proc Roy Soc B.

[4] Nijhout HF (1991). The Development and Evolution of Butterfly Wing Patterns.

[5] Ghiradella H, Locke M (1998). Hairs, bristles, and scales. Microscopic anatomy of invertebrates, Vol 11A: Insecta.

[6] Ghiradella H (2010). Insect cuticular surface modifications: scales and other structural formations. Adv Insect Physiol.

[7] Umebachi Y (1985). Papiliochrome, a new pigment group of butterfly. Zool Sci.

[8] Wilts BD, IJbema N, Stavenga DG (2014). Pigmentary and photonic coloration mechanisms reveal taxonomic relationships of the Cattlehearts (Lepidoptera: Papilionidae: *Parides*). BMC Evol Biol.

[9] Koch PB, Keys DN, Rocheleau T, Aronstein K, Blackburn M, Carroll SB (1998). Regulation of dopa decarboxylase expression during colour pattern formation in wild-type and melanic tiger swallowtail butterflies. Development.

[10] Koch PB, Behnecke B, ffrench-Constant RH (2000). The molecular basis of melanism and mimicry in a swallowtail butterfly. Curr Biol.

[11] Kinoshita S (2008). Structural colors in the realm of nature.

[12] Biró LP, Kertész K, Vertésy Z, Márk GI, Bálint Z, Lousse V (2007). Living photonic crystals: Butterfly scales - Nanostructure and optical properties. Mat Sci Eng C.

[13] Vukusic P, Sambles JR (2003). Photonic structures in biology. Nature.

[14] Kinoshita S, Yoshioka S, Fujii Y, Osanai M (2002). Photophysics of structural color in the *Morpho* butterflies. Forma.

[15] Michielsen K, Stavenga DG (2008). Gyroid cuticular structures in butterfly wing scales: biological photonic crystals. J R Soc Interface.

[16] Stavenga DG, Leertouwer HL, Wilts BD (2014). The colouration toolkit of the Pipevine Swallowtail butterfly, *Battus philenor*: thin films, papiliochromes, and melanin. J Comp Physiol A.

[17] Stavenga DG, Leertouwer HL, Wilts BD (2014). Colouration principles of nymphaline butterflies - thin films, melanin, ommochromes and wing scale stacking. J Exp Biol.

[18] Stavenga DG (2014). Thin film and multilayer optics cause structural colors of many insects and birds. Mat Today Proc.

[19] Stavenga DG, Leertouwer HL, Pirih P, Wehling MF (2009). Imaging scatterometry of butterfly wing scales. Opt Express.

[20] Yeh P (2005). Optical waves in layered media.

[21] Leertouwer HL, Wilts BD, Stavenga DG (2011). Refractive index and dispersion of butterfly scale chitin and bird feather keratin measured by interference microscopy. Opt Express.

[22] Nishikawa H, Iga M, Yamaguchi J, Saito K, Kataoka H, Suzuki Y (2013). Molecular basis of wing coloration in a Batesian mimic butterfly, *Papilio polytes*. Sci Reports.

[23] Schmidt K, Paulus H (1970). Die Feinstruktur der Flügelschuppen einiger Lycaeniden (Insecta, Lepidoptera). Z Morph Tiere.

[24] Ghiradella H (1991). Light and color on the wing: structural colors in butterflies and moths. Appl Optics.

[25] Bálint Z, Kertész K, Piszter G, Vertésy Z, Biró LP (2012). The well-tuned blues: the role of structural colours as optical signals in the species recognition of a local butterfly fauna (Lepidoptera: Lycaenidae: Polyommatinae). J R Soc Interface.

[26] Ingram AL, Parker AR (2008). A review of the diversity and evolution of photonic structures in butterflies, incorporating the work of John Huxley (The Natural History Museum, London from 1961 to 1990). Phil Trans R Soc B.

[27] Wasik BR, Liew SF, Lilien DA, Dinwiddie AJ, Noh H, Cao H (2014). Artificial selection for structural color on butterfly wings and comparison with natural evolution. Proc Natl Acad Sci U S A.

[28] Hidaka T, Yamashita K (1975). Wing color pattern as the releaser of mating behavior in the swallowtail butterfly, *Papilio xuthus L*. (*Lepidoptera: Papilionidae*). Appl Ent Zool.

